# Effects of probiotic supplementation on bone health in postmenopausal women: a systematic review and meta-analysis

**DOI:** 10.3389/fendo.2024.1487998

**Published:** 2024-11-01

**Authors:** Fang Wang, Wei Wei, Peng Ju Liu

**Affiliations:** Department of Clinical Nutrition, Peking Union Medical College Hospital, China Academic Medical Science and Peking Union Medical College, Beijing, China

**Keywords:** probiotic, postmenopausal women, osteoporosis, osteopenia, bone, bone turnover marker

## Abstract

**Context:**

The beneficial effects of probiotic supplementation on bone health in postmenopausal women require further validation.

**Objective:**

This study systematically reviewed and conducted a meta-analysis of randomized controlled trials (RCTs) to assess the relationship between probiotic supplementation and changes in bone mineral density (BMD) and bone turnover markers (BTMs) among postmenopausal women.

**Methods:**

A systematic search was conducted across four databases to retrieve data on lumbar spine BMD, hip BMD, collagen type 1 cross-linked C-telopeptide (CTX), receptor activator of nuclear factor-κB ligand (RANKL), osteocalcin (OC), osteoprotegerin (OPG), N-terminal propeptide of type 1 procollagen (P1NP), and bone-specific alkaline phosphatase (BALP) in postmenopausal women. Eligible RCTs were quantitatively analyzed using random-effects meta-analyses. Additional analyses, including subgroup, sensitivity, and meta-regression analyses, were performed.

**Results:**

Twelve RCTs involving 1183 postmenopausal women were included. Compared with the control group, postmenopausal women who received probiotic supplementation showed significantly greater BMD in both the lumbar spine (standardized mean difference [SMD] = 0.60, 95% confidence interval [CI] 0.14 to 1.05) and the hip (SMD = 0.74, 95%CI 0.15 to 1.33). Additionally, probiotic supplementation was associated with reduced levels of CTX (SMD = -1.51, 95%CI -1.88 to -0.41) and BALP (SMD = -1.80, 95%CI -2.78 to -0.81). No significant differences were found between the probiotic and control groups in terms of other BTMs. Subgroup analyses revealed that the increase in BMD due to probiotic supplementation was more significant in postmenopausal women with osteopenia than in those with osteoporosis. The meta-analysis results for both lumbar spine and hip BMD remained robust after conducting sensitivity analyses and meta-regressions.

**Conclusion:**

Supplementation with probiotics may increase BMD among postmenopausal women, with stronger evidence in women with osteopenia than osteoporosis. Further RCTs are suggested to confirm and refine these findings.

**Systematic review registration:**

https://www.crd.york.ac.uk/PROSPERO/, identifier CRD42024576764.

## Introduction

Osteoporosis is a common bone disease characterized by reduced bone mass and density, leading to an increased risk of fractures (1). Globally, it affects a significant proportion of the population, particularly among older adults. The prevalence of osteoporosis is higher in women than in men, with approximately one in three women over the age of 50 being affected (2). Postmenopausal women are particularly at risk for osteoporosis and fractures due to the natural decline in endogenous estrogen production, which is known to have a protective effect on bone mineral density (BMD). This decline in estrogen leads to a substantial reduction of bone mineral density (generally ranging from 2% to 5% per year) during the late perimenopausal period as well as in the first postmenopausal years (3). Moreover, the reduction in estrogen adversely impacts the bone’s microarchitecture, making the bones more susceptible to fractures.

Despite pharmaceutical interventions being available, adherence rates remain strikingly low, with less than 50% of patients continuing treatment beyond the first year (4, 5). This has been attributed to various factors, including a preference for alternative treatments and concerns over medication side effects (6). There is a clear trend toward seeking low-risk strategies to counteract the effects of osteoporosis, with dietary supplements like calcium and vitamin D gaining popularity. However, their impact on osteoporosis management may be less significant than initially thought (7), prompting a search for additional interventions (8).

Existing evidence has shown that there are remarkable changes in gut microbiota or its metablolites in postmenopausal women, and such changes are notably correlated with postmenopausal osteoporosis (PMO) (9–12). These correlations offer novel insights into the underlying mechanism of PMO and new strategies for treatment that could improve bone health in postmenopausal women.

Probiotics, gaining popularity as dietary interventions, are beneficial live microorganisms that can provide health advantages when consumed in sufficient quantities. There is a growing body of research highlighting the gut microbiota’s substantial influence on bone health through various interconnected mechanisms. This influence may involve the regulation of pro-inflammatory cytokines, which can increase bone resorption, the stimulation of intestine-derived estrogen production, the preservation of intestinal barrier integrity to prevent endotoxin translocation, and the increased production of short-chain fatty acids to inhibit osteoclast differentiation and promote the formation of osteoblastic cells as well as nutrient absorption essential for bone formation and maintenance (13–17). In animal models that mimic postmenopausal osteoporosis, supplementation with probiotics, including both *Lactobacillus* and *Bifidobacterium* species, has been demonstrated to significantly enhance BMD and bone volume in ovariectomized subjects (18). However, in human studies, particularly those focused on postmenopausal women, there exists a scarcity of comprehensive meta-analyses. The existing meta-analysis (19) is limited by a small number of randomized controlled trials (RCTs) included—specifically, only five studies (20–24)—and a focus on English-language publications, which restricts the generalizability of the findings.

Recently, additional available trials focusing on postmenopausal women (25–28), plus the prior literature from China (29–31), has expanded the available data, effectively doubling the number of studies considered compared to previous meta-analysis (19). This has prompted a new systematic review and meta-analysis of a broader range of RCTs. Consequently, we embarked on a systematic review and meta-analysis that encompassed a range of RCTs, with the objective to assess the potential skeletal benefits of probiotic interventions specifically in postmenopausal women.

## Materials and methods

This review was conducted in accordance with the PRISMA guidelines for reporting systematic reviews (32). The protocol has been registered in PROSPERO (https://www.crd.york.ac.uk/PROSPERO/, identifier: CRD42024576764).

### Data sources and searches

We systematically searched four electronic databases—*MEDLINE (PubMed)*, *Embase*, *Web of Science*, and *China National Knowledge Infrastructure (CNKI)*—from inception through 5 August 2024 for published RCTs evaluating the effects of probiotic supplementation (versus control or placebo) on bone mineral density (BMD) and bone turnover markers (BTMs) in postmenopausal women. We used the following search terms: ‘probiotics’, ‘probiotic*’, ‘lactobacillus’, ‘bifidobacterium’, ‘enterococcus’, ‘bone’, ‘bone mineral density’, ‘bone loss’, ‘bone turnover’, ‘osteoporosis’, ‘osteopenia’, ‘osteoporo*’, ‘osteopeni*’, ‘postmenopausal’, ‘post menopause’, and ‘postmenopause’ (see **Supplementary Table 1** for details on the search strategy). Reference lists of original trials were manually examined to obtain additional relevant data. The language was restricted to English and Chinese.

### Inclusion and exclusion criteria

The details regarding the PICOTS criteria are provided in **Supplementary Table 2**.

Inclusion criteria are as follows: 1) RCTs focusing on postmenopausal women; 2) the use of probiotic (multiple-strain or single-strain) supplementation as interventions and use of placebo (or control) as a comparison and consideration of the change of BMD and/or BTMs as outcomes, or trials with multiple interventions (e.g., coadministered probiotics and vitamin D or calcium) were eligible if the study groups differed only by the use of probiotics; 3) trials utilized dual-energy X-ray absorptiometry (DXA) for the measurement of BMD in the lumbar spine and hip at baseline and trial’s end. Concurrently, BTMs were identified via blood analysis at the same time points; 4) probiotic supplementation for at least 3 months and 5) original articles are written in English or Chinese. In addition, when results from a study population were reported more than once, the results with the longest follow-up time were utilized.

The following types of studies were excluded: 1) cross-sectional, cohort or case-control studies, reviews or meta-analyses, case reports, and animal or cell experiments; 2) articles only reporting protocols, editorials, comments, letters, conferences or abstracts of meeting presentations and 3) absence of expected data for meta-analysis.

### Data extraction

Two reviewers (FW and PJL) extracted independently the following information from each trial: the first author, year of publication, country, main participants’ characteristics (sample size, age, and body mass index), type of probiotics, intervention duration, other treatments, adherence with intervention, adverse effects, main outcomes including BMD in the lumbar spine and hip, collagen type 1 cross-l inked C-telopeptide (CTX), receptor activator of nuclear factor-κ B ligand (RANKL), osteocalcin (OC), osteoprotegerin (OPG), N-terminal propeptide of type 1 procollagen (P1NP), and bone-specific alkaline phosphatase (BALP). Additionally, the descriptions of the evidence of gut coloization for included studies after probiotic supplementation were presented in **Supplementary Table 3**. In the current study, BMD in the lumbar spine and hip were defined as the primary outcomes, while BTMs were utilized as secondary outcomes.

### Risk of bias assessment

Methodological quality was independently assessed by two investigators (W.W. and F.W.) by using the Cochrane Collaboration tool (33). All disagreements were resolved through consultation with a third investigator (P.J.L.). Bias in studies was appraised as low, high, or unclear, based on an evaluation of sequence generation, allocation concealment, participant and staff blinding, outcome assessor blinding, handling of incomplete data, selective outcome reporting, and other potential biases (**Supplementary Table 4**, **Supplementary Figures 1A, B**).

### Statistical analysis

Statistical analyses for this review were performed using STATA 14.0, and Review Manager 5.3 software. The impact of probiotic supplementation on bone status and BTMs was evaluated by examining the mean relative change from baseline to the conclusion of the intervention, alongside its standard deviation (SD). Direct usage of means and SDs of changes from baseline was prioritized; where not available, data were transformed using established methods (34–37). In cases where trials had multiple intervention arms of the same nature, they were combined into a single arm as per previous methods (36).

The pooled effects of the studies were expressed as standardized mean differences (SMD) with 95% confidence intervals (CIs). Heterogeneity among the studies was assessed using Cochrane’s Q test and quantified with the I^2^ statistic, with thresholds for low, moderate, and high heterogeneity set at <25%, 25%-50%, and >50%, respectively (38). A random-effects model was employed for calculating pooled effect measures in the presence of any heterogeneity (I^2^ > 0%). Sensitivity analyses were conducted to test the robustness of the results, involving the sequential omission of individual studies and further removal of studies with a high risk of bias to observe changes in heterogeneity. For the primary outcomes of lumbar spine and hip BMD, subgroup analyses were conducted to explore potential interactions based on the types of probiotic supplements, dosage of probiotic supplementation, intervention duration, geographical region, and participants’ baseline BMD indicators, including the presence or absence of osteoporosis (defined by a T-score of ≤ -2.5). Additionally, meta-regression analysis was utilized to determine if heterogeneity could be attributed to specific baseline characteristics such as age and BMI. Publication bias was assessed in meta-analyses with at least 10 studies using funnel plots and the Egger test (39). The trim-and-fill method was applied to identify and adjust for potential publication bias in the effect estimates.

## Results

### Search results

**Figure 1:**
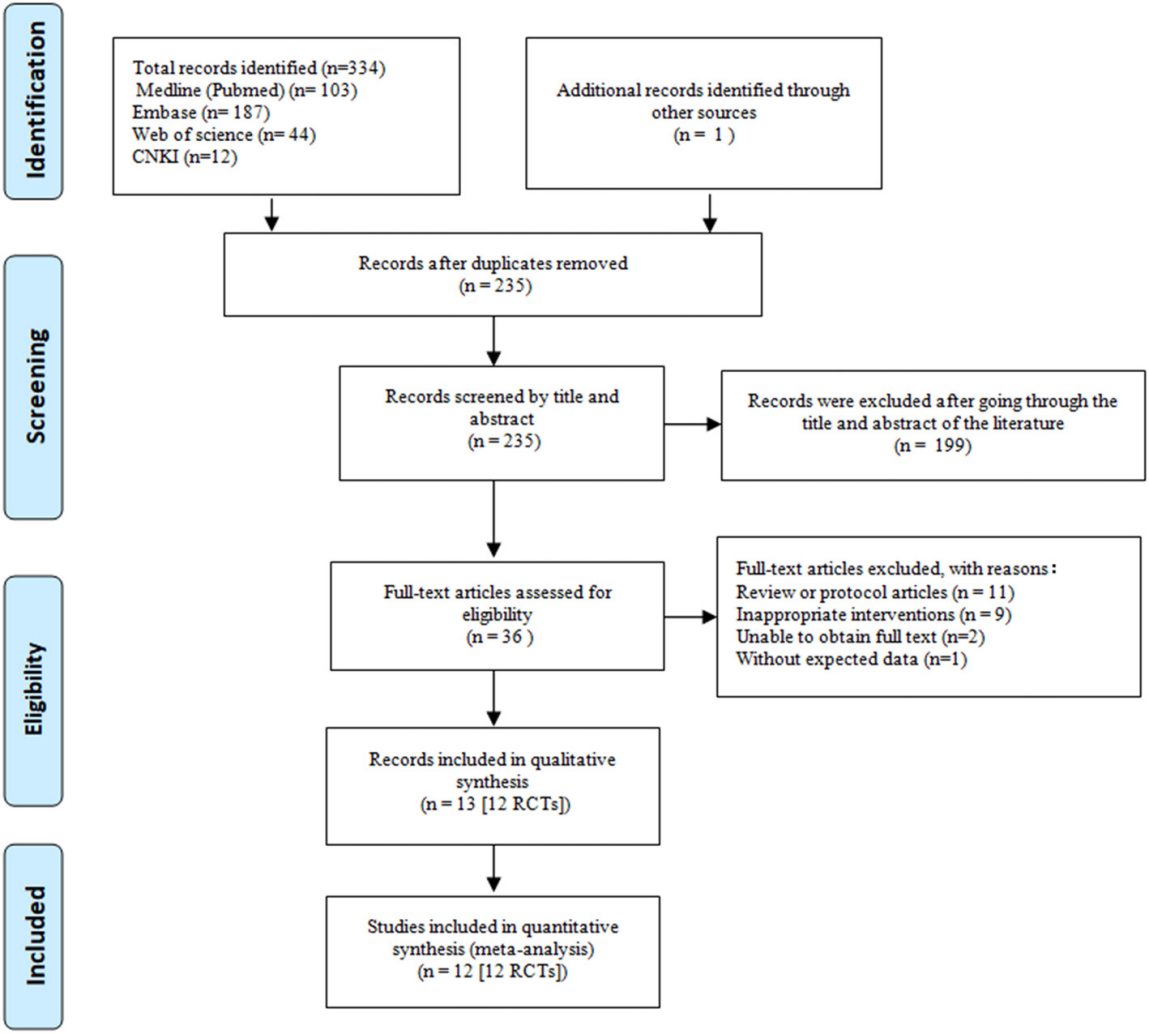
Flowchart of study selection.

Our initial search strategies across four databases yielded a total of 346 papers. After the removal of 112 duplicate records and screening the titles or abstracts of the remaining 234 records, we excluded 199 records that were obviously not relevant. The full text of the 35 eligible reports was read, which helped us to identify one additional article. Finally, we identified twelve RCTs that involved 1183 postmenopausal women (635 in the intervention group and 548 in the control group) as eligible for meta-analyses (20–31). A detailed overview of the selection process is provided in the PRISMA flow diagram in **Figure 1**.

### Characteristics of the included studies

The general characteristics of the included trials published between 2017 and 2024 were presented in **Table 1**. These studies were parallel-design, single-center trials conducted in the China (25, 29–31), Denmark (20), Iran (21), Japan (22), Poland (26), Sweden (23, 24, 27), and Thailand (28). Of these studies, four trials (22, 24, 26, 27) employed a single-strain probiotic as their intervention approach, in contrast to the rest that used multiple-strain probiotic formulations (20, 21, 23, 25, 28–31). The duration (3~24 months) of intervention varied across the studies, with three studies implementing interventions for a period of three months (25, 26, 28), five studies with interventions spanning six months (21, 22, 29–31), and four studies conducting interventions for a timeframe exceeding one year (20, 23, 24, 27). Additionally, eleven trials reported the results of lumbar spine BMD (20–27, 29–31), and ten studies described the results of hip BMD (20–27, 29, 30). According to the dosage of probiotics, seven studies were defined as the high-dose group (≥ 1 x 10^9 CFU/d) (20–27) and three as the low-dose group (< 1 x10^8 CFU/d) (29–31).

**Table 1:** Characteristics of included randomized controlled trials in the meta-analysis.

Study (Author, year)	Country	Sample size (C/I group)	Blinding	Mean age, years (mean ± SD)	BMI kg/m^2^ (mean ± SD)	Descriptions of intervention	Comparison intervention	Duration (months)	Adherence with intervention	Outcomes	adverse events
Jansson (2019)	Sweden	123/126	Double blind	C: 58.1± 4.3 I: 59.1 ± 3.8	C: 23.9 ± 2.6 I: 24.2 ± 2.7	Three lactobacillus strains: L. paracasei 8700:2 (DSM 13434), L. plantarum heal 9(DSM 15312), and L. plantarum heal 19(DSM 15313)	Placebo	12	NR	BMD (lumbar spine and total hip)	The number of adverse events considered to be related to the treatment were similar between the groups (24% in Lactobacillus and 26% in placebo). No treatment-related serious adverse events reported
Takimoto (2018)	Japan	30/31	Double blind	C: 57.8± 5.4 I: 57.5 ± 4.3	C: 23.9 ± 2.6 I: 24.2 ± 2.7	Probiotic Bacillus subtilis C-3102 (C-3102)	Placebo	6	The overall mean compliance rate was 99.5% ± 0.1% (placebo = 99.6% ± 0.2%, C-3102 = 99.5% ± 0.2%)	BMD (lumbar spine and total hip)	No adverse effects were reported during the study period
Lambert (2017)	Denmark	40/38	Double blind	C: 62.9± 1 I: 60.8 ± 1.1	C: 26.7 ± 0.8 I: 24.8 ± 0.6	Lactic acid bacteria and soffavones Other treatments: twice daily red clover extract (RCE) plus vitamin and mineral tablets containing 1040 mg Ca, 487 mg Mg, and 25 mg vitamin D/d	Placebo	12	The mean compliance rate was 96.44% ± 0.40%. There were no significant intergroup differences in compliance rates.	BMD (lumbar spine and hip [femoral neck]), CTX, OPG, RANKL, OC	Three participants (one in control group and two in intervention group) dropped out of the study due to gastrointestinal issues, and there was no significant difference between groups
Jafarnejad (2017)	Iran	21/20	Double blind	C: 57.3± 0.7 I: 68.9 ± 0.7	C: 23.8 ± 0.4 I: 24.9 ± 0.4	Multispecies probiotic supplement (GeriLact capsule): L. casei, Biffdobacterium longum, L. acidophilus, L. rhamnosus, L. bulgaricus, Biffdobacterium breve and Streptococcus thermophilus Other treatments: 500 mg calcium plus 200 IU vitamin D daily	Placebo+other treatments identical to those in the intervention group	6	NR	BMD (lumbar spine and hip), CTX, RANKL, OPG, OC, BALP	There were no significant adverse effects reported directly attributed to the treatment
Nilsson (2018)	Sweden	36/32	Double blind	C: 76.3± 1.1 I: 76.4 ± 1.0	C: 25.3 ± 3.3 I: 25.5 ± 3.5	Freeze-dried L. reuteri 6475 (BioGaia AB, Stockholm, Sweden)	Placebo	12	NR	BMD (lumbar spine and total hip), CTX, BALP	Adverse events considered to be related to the treatment were similar between the groups (40% in L. reuteri 6475 and 44% in placebo)
Vanitchanont (2024)	Thailand	20/20	Double blind	C: 64.1 ± 3.6 I: 62 ± 5.1	C: 24.2 ± 2.8 I: 23.4 ± 3.8	Lactobacillus reuteri GL-104, Lactobacillus paracasei MP-137, Lactobacillus rhamnosus MP108, Lactobacillus rhamnosus F-1, Lactobacillus rhamnosus BV77, Biffdobacterium animalis ssp. lactis CP-9, Biffdobacterium longum ssp. longum OLP-01, and Bacillus coagulans Other treatments: at least 1200 mg of calcium daily and 20,000 IU of vitamin D2 per week	Placebo+other treatments identical to those in the intervention group	3	NR	CTX, P1NP	Four participants in the placebo group and two in the multispecies probiotic group reported adverse reactions during the study period. There were no significant differences between groups in these adverse reactions.
Gregori (2024)	Sweden	79/160	Double blind	C: 55(53-56) High-dose: 55(52-56) Low-dose: 55(53-56)	C:23.7(21.4-28.3) High-dose:23.9(22.1-27.5) Low-dose:24.5(21.9-27.9)	High-dose: L reuteri 6475 (BioGaia AB) (5 × 10^9^ colony-forming units) Low-dose: L reuteri 6475 (BioGaia AB) (5 × 10^8^ colony-forming units) Other treatments: 200 IU of cholecalciferol per day	Placebo+other treatments identical to those in the intervention group	24	Overall, mean (SD) adherence to the study product was high, ranging from 87.5% (24.6%) in the high-dose L reuteri group to 93.6% (12.9%) in the placebo group	BMD (lumbar spine and total hip), CTX, P1NP	No significant adverse effects were observed.
Harahap (2024)	Poland	32/32	Double blind	Overall: 45-70	C: 28.6 ± 4.4 I: 25.3 ± 4.8	L. acidophilus UALa-01	Placebo	3	NR	BMD (lumbar spine and hip [femoral neck]), PINP, CTX, BALP	A significant increase in glucose concentration was observed in the probiotic group
Zhao (2023)	China	20/20	Double blind	C:61.6 ± 7.9 I: 62.8 ± 6.0	C: 23.4 ± 2.5 I: 23.1 ± 2.2	Bifdobacterium animalis subsp. lactis Probio-M8, Probio-M8 Other treatments: daily 600 mg of calcium and 0.25µg of calcitriol	Placebo+other treatments identical to those in the intervention group	3	NR	BMD (lumbar spine and hip [femoral neck]), PINP, CTX, OC	NR
Li (2021)	China	73/73	Unclear	C: 69.8 ± 21.5 I: 68.2 ± 22.4	C: 24.9 ± 7.4 I: 26.3 ± 8.4	Bifidobacterium quadruple viable bacteria tablets 0.5 g Tid + oral alendronate sodium 10 mg Qd + subcutaneous or intramuscular injection of salmon calcitonin 50 IU Qd.	Oral alendronate sodium 10 mg Qd + subcutaneous or intramuscular injection of salmon calcitonin 50 IU Qd.	6	NR	BMD (lumbar spine and hip [femoral neck]), CTX, OC, BALP	There were no significant differences between groups in adverse reactions.
Guo (2020)	China	24/30	Double blind	C: 63.4 ± 5.7 I: 61.9 ± 6.4	C: 23.9 ± 3.2 I: 23.6 ± 3.4	Dry Probio-M8 lactic acid bacteria	placebo	6	NR	BMD (lumbar spine and hip [femoral neck]), CTX, OC	NR
Zhang (2018)	China	50/53	Unclear	C:58.6 ± 7.5 I:56.8 ± 6.2	C:25.9 ± 3.7 I:25.9 ± 3.0	Bifidobacterium quadruple viable bacteria tablets 0.5 g Tid+ calcium carbonate 600mg/d+vitamin D3 (125IU/d)	calcium carbonate 600mg/d+vitamin D3 125IU/d	6	NR	BMD (lumbar spine), CTX, BALP	NR

C, control; CTX, collagen type 1 cross-l inked C-telopeptide; I, intervention; BMD, bone mineral density; RANKL, Receptor activator of nuclear factor-κ B ligand; OC, osteocalcin; OPG, osteoprotegerin; PINP, N-terminal propeptide of type I procollagen; BSAP, bone-speciﬁc alkaline phosphatase; NR, not reported; BALP, bone-specific alkaline phosphatase.

### Main outcomes

#### Effects of probiotic supplementation on BMD

**Figure 2:**
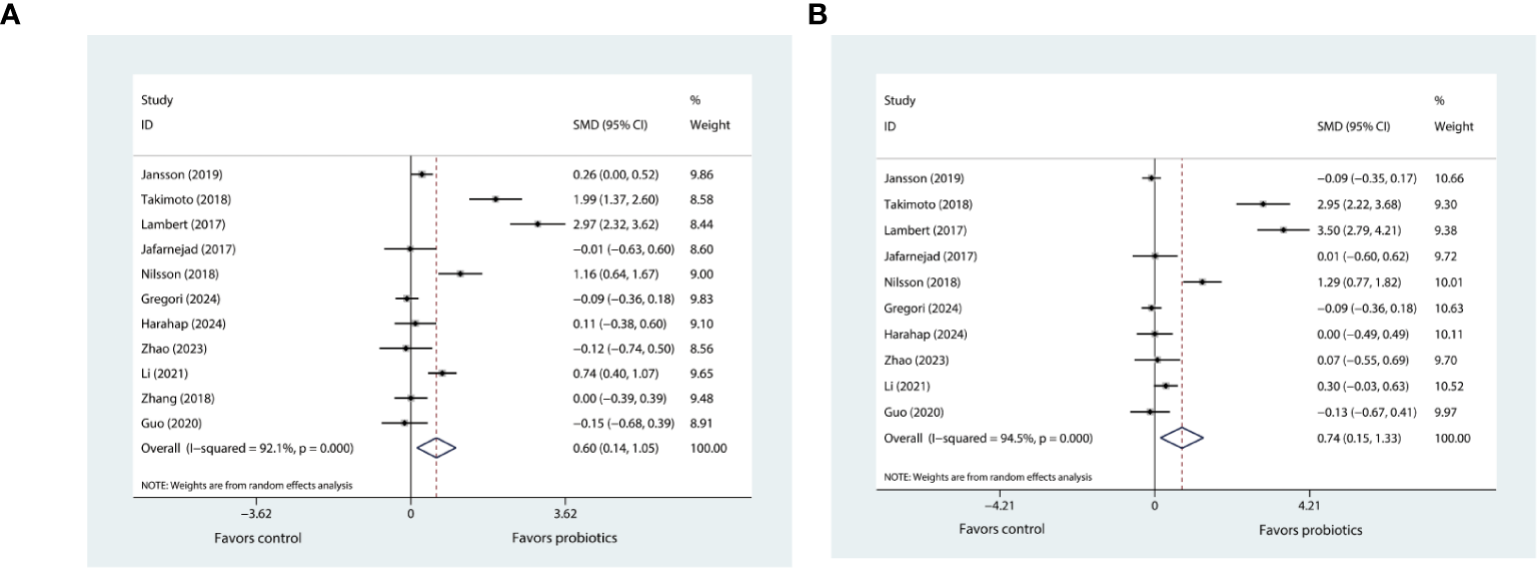
**(A)** Forest plot depicting the overall effect of probiotics on lumbar spine BMD. **(B)** Forest plot depicting the overall effect of probiotics on hip BMD.

The meta-analysis investigating the effects of probiotics on BMD in the lumbar spine involved eleven trials (20–27, 29–31), while that concerning the hip BMD comprised ten trials (20–27, 29, 30). The pooled results using the random effects model showed that probiotic supplementation had a positive effect on both lumbar spine BMD (SMD=0.60, 95%CI [0.14, 1.05], P=0.01; I^2^ = 92.1%; **Figure 2A**) and hip BMD (SMD=0.74, 95%CI [0.15, 1.33], P=0.013; I^2^ = 94.5%; **Figure 2B**) when compared with control.

### Heterogeneity, subgroup, sensitivity analyses and meta-regressions

For the primary outcomes included in the meta-analysis, there was obvious heterogeneity (*I^2^* > 50%). Subsequently, subgroup, sensitivity analyses and meta-regressions were performed to try to identify potential sources of heterogeneity.

The sensitivity analysis for lumbar spine BMD showed that the *I²* statistic did not fall below 50% after excluding any single trial from the eleven, and the hip BMD analysis also resulted in a similar finding. In addition, the *I²* statistic for both lumbar spine and hip BMD continued to exceed 50% even after the removal of trials that were reported in the Chinese language. Despite this, the meta-analysis results for both lumbar spine and hip BMD were robust.

Subgroup analyses revealed that probiotic supplementation, regardless of whether it involves single- or multiple-strain formulations, resulted in significant increases in both lumbar spine and hip BMD in trials that specifically included postmenopausal women with osteopenia. This enhancement in BMD was notably more pronounced in comparison to trials involving women who have osteoporosis (characterized by a T-score of more than or equal to -2.5) (**Tables 2**, **3**). Furthermore, our results indicated that high-dose supplementation of probiotics could improve both lumbar spine and hip BMD more effectively than low-dose supplementation (**Tables 2**, **3**). In addition, our findings showed that extended probiotic supplementation (at least one year) indicated greater benefits for enhancing lumbar spine BMD (SMD=1.03, 95%CI [0.06, 2.00], P=0.037; *I^2^ =* 96.4%) compared to supplementation regimens that last for six months or less (**Table 2**). Meta-regressions did not reveal any significant correlation between the effects of probiotics on BMD and the age and BMI of postmenopausal women (**Supplementary Figures 2A, B**).

**Table 2:** Results of subgroup analyses of the effects of probiotics on lumbar spine BMD.

Subgroup		Lumbar spine BMD			
		*Effect size*	*95% CI*	*I^2^*	*P value*
T score < -2.5	No (n = 8)	0.76	0.17, 1.36	93.9%	0.012
	Yes (n = 3)	0.19	-0.46, 0.84	80.9%	0.559
Intervention duration	≤ 6 months (n = 7)	0.36	-0.14, 0.86	86.1%	0.155
	> 6 months (n = 4)	1.03	0.06, 2.00	96.4%	0.037
Types of probiotics	Single-strain (n = 4)	0.77	-0.13, 1.66	93.7%	0.095
	Multiple-strain (n = 7)	0.51	-0.08, 1.10	92.3%	0.091
Region	Asia (n =6)	0.40	-0.18, 0.98	88.0%	0.173
	Europe (n = 5)	0.84	0.05, 1.63	95.2%	0.037
Dosage of probiotics	≥1 x 10^9 CFU/d (n = 8)	0.76	0.13, 1.39	93.9%	0.018
	< 1 x 10^8 CFU/d (n = 3)	0.22	-0.35, 0.79	82.5%	0.451

BMD, bone mineral density; CFU, colony forming unit; CI, confidence interval.

**Table 3:** Results of subgroup analyses of the effects of probiotics on hip BMD.

Subgroup		Hip BMD			
		*Effect size*	*95% CI*	*I^2^*	*P value*
T score < -2.5	No (n = 7)	1.04	0.19, 1.89	96.3%	0.016
	Yes (n = 3)	0.17	-0.09, 0.42	0.0%	0.201
Intervention duration	≤ 6 months (n = 6)	0.50	-0.21, 1.22	91.0%	0.165
	> 6 months (n = 4)	1.10	-0.02, 2.22	97.2%	0.054
Types of probiotics	Single-strain (n = 4)	1.00	-0.15, 2.16	95.9%	0.088
	Multiple-strain (n = 6)	0.58	-0.20, 1.35	94.4%	1.144
Region	Asia (n =5)	0.61	-0.26, 1.50	92.5%	0.169
	Europe (n = 5)	0.87	-0.03, 1.78	96.3%	0.059
Dosage of probiotics	≥1 x 10^9 CFU/d (n = 7)	1.04	0.19, 1.89	96.3%	0.016
	< 1 x 10^8 CFU/d (n = 3)	0.17	-0.09, 0.42	0.0%	0.201

BMD, bone mineral density; CFU, colony forming unit; CI, confidence interval.

### Effects of probiotic supplementation on bone turnover markers and sensitivity analyses

#### CTX, BALP, and P1NP

**Figure 3:**
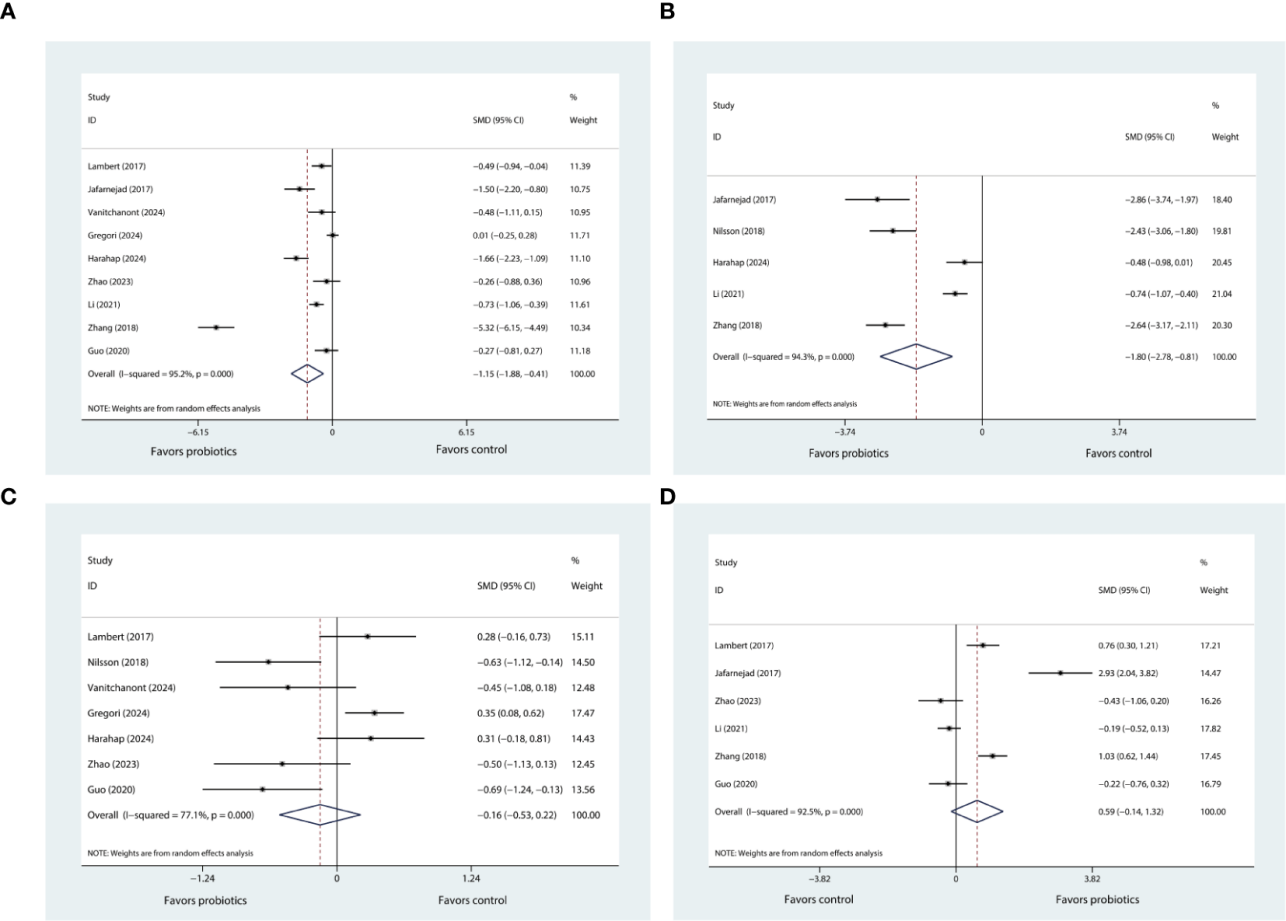
**(A)** Forest plot depicting the overall effect of probiotics on CTX. **(B)** Forest plot depicting the overall effect of probiotics on BALP. **(C)** Forest plot depicting the overall effect of probiotics on P1NP. **(D)** Forest plot depicting the overall effect of probiotics on OC.

According to data pooled from nine eligible trials (20, 21, 25–31), when compared with control, probiotic supplementation significantly reduced CTX levels (SMD= -1.51, 95%CI [-1.88, -0.41], P= 0.002; **Figure 3A**). The degree of heterogeneity was high (*I^2^ =* 95.2%).

There were five eligible trials reporting the results of BALP (21, 24, 26, 30, 31). Upon consolidating the data from these studies, it was determined that the supplementation with probiotics was associated with a significant reduction in BALP levels (SMD= -1.80, 95%CI [-2.78, -0.81], P < 0.001; I^2^ = 94.3%; **Figure 3B**) when compared to the control group.

The sensitivity analysis, in which we sequentially removed each of the nine trials from the meta-analysis, showed that the *I²* statistic did not decrease below 50%. Despite this persistent heterogeneity, the overall meta-analysis results for CTX-1maintained their robustness. The similar findings were observed in the sensitivity analysis of BALP.

Based on the pooled data from seven trials (20, 24–29), our results indicated that probiotic supplementation did not exert any significant influence on P1NP levels (SMD= 0.59, 95%CI [-0.14, 1.32], P= 0.112; I^2^ = 92.5%; **Figure 3C**) in comparison to control group. After conducting sensitivity analysis, it was observed that the results of meta-analysis for P1NP remained stable and did not show any significant alteration. Also, the heterogeneity across the studies, as indicated by the I² statistic, remained high, exceeding 50%.

#### OC, OPG and RANKL

Six trials (20, 21, 25, 29–31) reported changes in OC levels before and after the intervention. Pooled analysis showed that probiotic supplementation did not exert any significant influence on OC levels (SMD= -0.16, 95%CI [-0.53, 0.22], P= 0.416; *I^2^ =* 77.1%; **Figure 3D**) when compared to control. Through further sensitivity analysis, we did not observe any change in the meta-analysis results or heterogeneity. Only two trials reported the results pertaining to OPG (20, 21) (**Supplementary Figure 3A**) and RANKL (20, 21) (**Supplementary Figure 3B**), respectively. The pooled results indicated that use of probiotic supplementation had no significant effect on these two markers.

### Publication bias

Potential publication bias was detected using funnel plots and the Egger test. The funnel plots of lumbar spine BMD (**Supplementary Figure 4A**) and hip BMD (**Supplementary Figure 4B**) were displayed in the **Supplementary Materials**. Funnel plots were not created for the other markers as there were fewer than ten included trials in their meta-analyses (39). The Egger test results revealed publication bias for the effects of probiotic supplementation on hip BMD (t=2.42, P=0.042; **Supplementary Figure 5B**), while the results regarding lumbar spine BMD did not show publication bias (t=1.52, p=0.162; **Supplementary Figure 5A**). We used the trim-and-fill method to detect and adjust for publication bias regarding the results of hip BMD, but the updated overall effect estimate did not show significant changes (z=2.945, P=0.003; **Supplementary Figure 5C**). Consequently, more studies with large number are further needed.

## Discussion

### Main findings

This meta-analysis of twelve RCTs involving 1183 postmenopausal women sheds new light on the influence of probiotic supplementation on bone health in this demographic. The analysis revealed that supplementation with probiotics, especially at a dose of at least 1 x 10^9 CFU per day, positively impacted bone health, with stronger evidence in women with osteopenia than osteoporosis, as indicated by improvements in lumbar spine and hip BMD. Additionally, probiotic supplementation correlated with reduced levels of CTX and BALP, pointing to a potential anti-osteoporotic effect of probiotics. Despite significant heterogeneity across the included studies, the findings are supported by objective primary and secondary outcome measures and a robust random-effects analysis model. The credibility of the results is further supported by the stability of the meta-analysis outcomes following sensitivity analysis.

**Figure 4:**
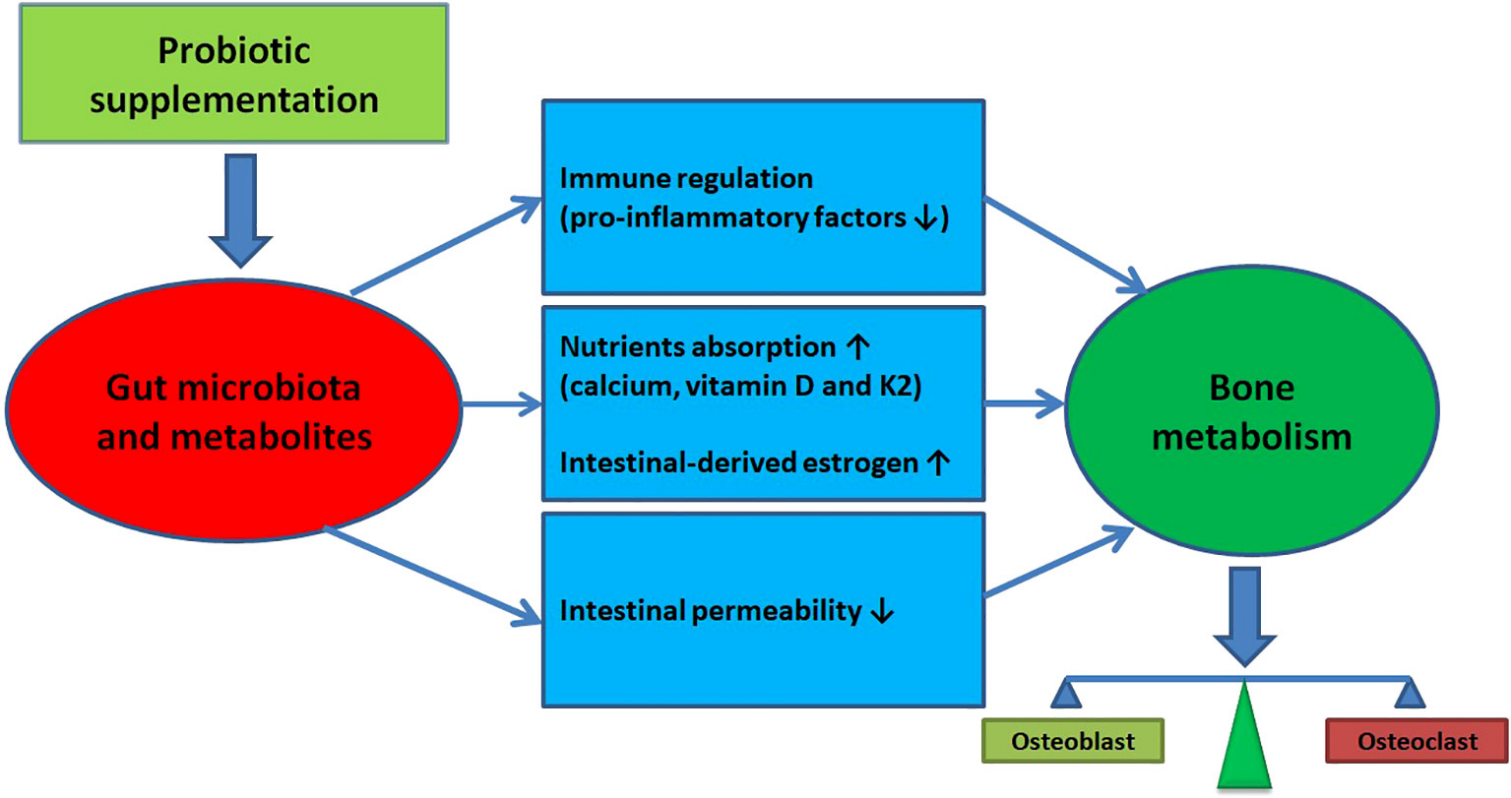
The potential impact of gut microbiota regulation on promoting bone health.

Postmenopausal women are disproportionately affected by osteoporosis due to estrogen deficiency (40, 41). The current understanding of osteoporosis is not yet sufficient to develop pharmaceuticals capable of completely preventing or stopping the disease’s progression (42). However, research has shown a link between the gut microbiota and bone mass reduction, as well as osteoporosis prevalence (13–16, 43, 44). The gut microbiota is believed to influence bone metabolism by affecting the balance between osteoclast and osteoblast activity, thereby impacting the host’s metabolism and immune system (9, 10, 45) (The diagram illustrating the potential impact of gut microbiota regulation on promoting bone health is presented in **Figure 4**). This has led to the exploration of modulating the intestinal microbiome, such as through probiotic supplementation, as a treatment for osteoporosis or osteopenia in postmenopausal women (20–31). However, the effects of probiotics on the bone health in postmenopausal women are still inconsistent.

To our knowledge, only one previous meta-analysis has specifically examined the effects of probiotic supplementation on bone health in postmenopausal women (19). That study found positive effects on BMD in the spine but no observed benefits in the hip region. Another meta-analysis (46) evaluated the impact of probiotics on bone health among postmenopausal women and other individuals suffering from senile or diabetic osteoporosis, and it reported similar findings regarding BMD in the lumbar spine and hip among postmenopausal women as Yu et al.’s study (19). The discrepancy in the effects of probiotics on BMD in different areas among postmenopausal women may be due to the small sample size of studies included in these two meta-analyses (19, 46). Our current meta-analysis reaffirms the previous findings on lumbar spine BMD and further expands on these results by demonstrating positive effects of probiotics on hip BMD as well.

Additionally, subgroup analyses were conducted and revealed that the improvement in BMD was more pronounced in postmenopausal women with mild bone loss (osteopenia) (20–24, 26–31), when treated with probiotics, as opposed to those with osteoporosis (25, 29, 30). This may be due to the limited inclusion of studies in the osteoporosis group. Therefore, further research is needed on probiotic intervention for postmenopausal osteoporosis. When the included trials were divided into subgroups according to intervention duration (≤6 months or > 12 months), we found extended durations of probiotic intervention (> 12 months) (20, 23, 24, 27), in contrast to brief periods of treatment, demonstrated superior enhancement in lumbar spine BMD (P=0.037), suggesting that longer-term probiotic supplementation may yield more benefits in bone health. Additionally, the improvement in hip BMD did not reach statistical significance (SMD= 1.10, 95%CI [-0.02, 2.22], P=0.054). We also conducted subgroup analysis based on study region (Europe and Asia), and found that probiotics appear to be more effective in enhancing lumbar spine BMD in postmenopausal women from Europe compared to those from Asian backgrounds (21, 22, 25, 28–31). This observation could be attributed to potential differences in gut microbiome composition, genetic factors, lifestyle, or dietary habits between these populations, although further research is needed to clarify these regional disparities in response to probiotic treatment. Of note, our subgroup analysis also suggested that there was no significant difference in the impact on BMD between single- and multiple-strain probiotics.

CTX and P1NP are both widely recognized bone turnover markers (BTMs) in clinical use, with CTX indicating bone resorption and P1NP indicating bone formation (47). Our study’s findings that probiotic supplementation can lead to a reduction in CTX levels are in line with two previous meta-analyses (19, 46), neither of which reported the effects of probiotics on P1NP due to the limited data. By synthesizing our findings on both P1NP and CTX, it is clear that the mechanism through which probiotic supplementation may help in preventing bone loss is likely due to its effect on inhibiting bone resorption by suppressing osteoclast activity (22). BALP, a marker traditionally associated with osteoblast proliferation, is recognized as a bone formation indicator (45). However, there is a growing body of evidence suggesting that BALP should be reclassified as a marker of bone turnover rather than solely bone formation (48, 49). In our study, it was found that levels of BALP could be decreased by supplementation with probiotics. This is consistent with the findings of other studies, which have reported a decrease in BALP following the consumption of symbiotic products (50) or specific probiotic strains such as Lactobacillus reuteri (51). These collective findings suggest that the classification of BALP as a bone formation marker may need to be reconsidered in light of its association with the broader process of bone turnover. However, the meta-analysis by Yu et al. showed no significant changes in BALP (19). This is most likely due to the limited number of studies included.

In addition our study also examined other bone turnover markers, including OC, OPG, and RANKL. Our findings are in line with previous meta-analyses (19, 46), which did not find any significant differences in the levels of these markers between postmenopausal women who received probiotics and those in the control group. However, it is important to highlight a preclinical study that demonstrated the supplementation of heat-killed *Lacticaseibacillus paracasei GMNL-653* could lead to a specific reduction in the *mRNA* level of RANKL in ovariectomized mice; Whole-genome sequencing and comparative genomics analysis indicated that genes associated with the transport and metabolism of carbohydrates, as well as the biogenesis of cell walls, membranes, and envelopes, might play a role in the anti-osteoporotic effects of GMNL-653 (52). This observation prompts the hypothesis that the impact of different probiotic strains on bone turnover markers might vary. To substantiate these preliminary findings, further research is essential to explore the potential variability in effects among different strains of probiotics on BTMs.

### Limitations and strengths

Our study has several limitations. Firstly, there is high heterogeneity between the included studies, although random-effects model was used to calculate the results, complemented by suitable subgroup analyses and meta-regressions. Secondly, we had to calculate SMD rather than the weighted mean difference due to the inconsistent units describing BMD change among the included studies. Third, in several trials (20–22, 25, 27, 28, 30, 31), co-interventions were used as treatment methods, rather than probiotics alone. This might lead to potential bias; however, after we conducted sensitivity analysis, the robustness of the meta-analysis findings was maintained. Lastly, based on the current trials included, it was not possible for us to discern which probiotic strains specifically improve BMD or BTMs.

On the other hand, this study also has several strengths. Firstly, most of the included trials were of high quality. Secondly, we performed adequate subgroup analyses; sensitivity analysis and meta-regressions were also conducted to minimize heterogeneity between the included studies. Thirdly, compared with previous meta-analyses and reviews, this meta-analysis includes a larger number of trials and a wider population.

## Conclusion

Our systematic review and meta-analysis found that probiotic supplementation in postmenopausal women was associated with improved BMD in the lumbar spine and hip, with stronger evidence in women with osteopenia than osteoporosis. This suggests that probiotic supplementation may serve as an alternative approach to decelerate bone mass deterioration in postmenopausal women with osteopenia. In addition, administration of probiotics could decrease levels of CTX and BALP. In the future, more research is needed to validate these findings, and specific strains beneficial for bone health in postmenopausal women need to be further explored.

## Data Availability

The original contributions presented in the study are included in the article/**Supplementary Material**, further inquiries can be directed to the corresponding author.
