# Supplementary material for: Effects of probiotic supplementation on bone health in postmenopausal women: a systematic review and meta-analysis
Source: Front Endocrinol (Lausanne). 2024 Nov 1;15:1487998. doi: 10.3389/fendo.2024.1487998 (PMC11563942; doi:10.3389/fendo.2024.1487998)
Supplement: Supplementary file 1 [file DataSheet1.docx]

**Effects of probiotic supplementation on bone health in postmenopausal women: A systematic review and meta-analysis**

Fang Wang^1^, Wei Wei^1^, and Peng Ju Liu^1^

^1^ Department of Clinical Nutrition, Peking Union Medical College Hospital, China Academic Medical Science and Peking Union Medical College, Beijing, 100730, PR China

Emails:

Fang Wang: [yours.fang@hotmail.com](mailto:yours.fang@hotmail.com)

weiwei@pumch.cn

Peng Ju Liu: [lpjjia@126.com](mailto:lpjjia@126.com)

***Correspondence:** Peng Ju Liu, Department of Clinical Nutrition, Peking Union Medical College Hospital, NO. 1 Shuaifuyuan, Dongcheng District, Beijing, China. Email: [lpjjia@126.com](mailto:lpjjia@126.com), Tel: 861069155553

***CONTENTS***

*Supplementary Table S1: The search strategy of the systematic review.*

*3-5*

*Supplementary Table S2: Summary of the PICTOS criteria used to identify studies for inclusion and exclusion*

*6*

*Supplementary Table S3: Evidence of gut colonization*

*7*

*Supplementary Table S4: Risk of bias assessment for included randomized controlled clinical trials.*

*8*

*Supplementary Figure S1A: Risk of bias in seven domains for all included studies*

*9*

*Supplementary Figure S1B: Risk of bias assessment across seven domains for each included study*

*10*

*Supplementary Figure S2A: Meta-regression of age on lumbar spine and hip BMD*

*Supplementary Figure S2B: Meta-regression of BMI on lumbar spine and hip BMD*

*11-14*

*Supplementary Figure S3A: Forest plots depicting the effect of probiotics on osteoprotegerin (OPG)*

*15*

*Supplementary Figure S3B: Forest plots depicting the effect of probiotics on receptor activator of nuclear factor-κB ligand (RANKL)*

*16*

*Supplementary Figure S4A: Funnel plot for lumbar spine BMD*

*17*

*Supplementary Figure S4B: Funnel plot for hip BMD*

*18*

*Supplementary Figure S5A: Egger’s publication bias plot of lumbar spine BMD*

*19*

*Supplementary Figure S5B: Egger’s publication bias plot of hip BMD*

*20*

*Supplementary Figure S5C: Publication bias regarding the results of hip BMD after the trim-and-fill method*

*21*

***Supplementary Table S1. The search strategy of the systematic review* MEDLINE（PUBMED）— from 1946**

1. Probiotics/
2. probiotic*.mp.
3. exp Lactobacillus/
4. lactobacillus.mp.
5. exp Bifidobacterium/
6. Bifidobacterium.mp.
7. exp Enterococcus/
8. enterococcus.mp.
9. Prebiotics/
10. prebiotic*.mp.
11. Synbiotics/
12. synbiotic*.mp.
13. OR/1-18
14. Postmenopausal period/
15. postmenopause.mp.
16. postmenopausal.mp.
17. post menopause.mp.
18. OR/14-17
19. bone.mp.
20. Bone loss.mp.
21. Bone loss*.mp.
22. Osteoporosis/
23. Osteoporo*.mp.
24. Osteopeni*.mp.
25. Bone mineral density.mp.
26. Bone turnover.mp.
27. OR/19-26
28. 13 AND 18 AND 27

**Embase— from 1947**

=============================================================

1. ‘probiotic agent’/
2. ‘probiotic*’.ab,ti,kw.
3. exp ‘Lactobacillus’/
4. ‘lactobacillus’.ab,ti,kw
5. exp ‘Bifidobacterium’/
6. ‘Bifidobacterium’.ab,ti,kw
7. exp ‘Enterococcus’/
8. ‘enterococcus’.ab,ti,kw
9. ‘prebiotic agent’/
10. ‘prebiotic*’/ab,ti,kw
11. ‘Synbiotic agent’/
12. ‘synbiotic*’.ab,ti,kw
13. OR/1-12
14. ‘Osteoporosis’/
15. ‘Osteopeni*’.ab,ti,kw.
16. ‘Osteoporo*’.ab,ti,kw.
17. ‘Bone’.ab,ti,kw
18. ‘Bone loss’.ab,ti,kw
19. ‘Bone loss*’.ab,ti,kw.
20. ‘Bone mineral density’.ab,ti,kw.
21. ‘Bone turnover’.ab,ti,kw.
22. OR/14-21
23. ‘Postmenopause’. ab,ti,kw.
24. ‘Postmenopausal’ .ab,ti,kw.
25. ‘Post menopause’ .ab,ti,kw.
26. OR/23-25
27. 13 AND 22 AND 26

**Web of Science**

**============================================================**

1. (((((TS=(probiotic*)) OR TS=(lactobacillus)) OR TS=(bifidobacterium)) OR TS=(enterococcus)) OR TS=(prebiotic*)) OR TS=(synbiotic*)
2. ((TS=(postmenopause)) OR TS=(postmenopausal)) OR TS=( post menopause)
3. **(((((((TS=(Osteoporo*)) OR TS=(Osteopeni*)) OR TS=(Bone loss)) OR TS=(Bone loss*)) OR TS=(Bone mineral density)) OR TS=(Bone turnover)) OR TS=(bone)**
4. #1 AND #2 AND #3

**Abbreviations/symbols: /**, Medical Subject Heading (MeSH) for MEDLINE; *, any character; mp., multipurpose (searches several fields including the MEDLINE title, original title, abstract, subject heading, name of substance, and registry word fields).

***Supplementary Table S2.*** ***Summary of the PICOTS criteria used to identify studies for inclusion and exclusion***

| **Parameter** | **Description** |
| --- | --- |
| ***Population*** | Postmenopausal women. |
| ***Intervention*** | The use of probiotic (multiple-strain or single-strain) supplementation as interventions, or multiple interventions (e.g., co-administered probiotics and vitamin D or calcium) as long as the study groups differed only by the use of probiotics |
| ***Comparison*** | Use of placebo (or control) as a comparison |
| ***Outcome*** | **Primary outcomes:**   1. Lumbar spine bone mineral density 2. Hip bone mineral density   **Secondary outcomes:**   1. Collagen type 1 cross-l inked C-telopeptide 2. Osteocalcin 3. Osteoprotegerin 4. Bone-specific alkaline phosphatase 5. N-terminal propeptide of type 1 procollagen 6. Receptor activator of nuclear factor-κB ligand |
| ***Time*** | The duration of the study was at least 3 months |
| ***Study design*** | Randomized controlled trial (parallel) |
| ***exclusion*** | 1) cross-sectional, cohort or case-control studies, reviews, meta-analyses, case reports, and animal or cell experiments;  2) trials without expected outcomes;  3) unable to extract the relevant data due to the presentation format of them;  4) trials with an intervention duration of less than 3 months;  5) articles only reporting protocols, editorials, comments, letters, conferences or abstracts of meeting presentation. |

***Supplementary Table S3:*** ***Evidence of gut colonization***

| Study | Evidence of gut colonization |
| --- | --- |
| Jansson (2019) | Without reporting the evidence of gut colonization |
| Takimoto (2018) | The relative abundance of genus Biffdobacterium signiffcantly increased at 12 weeks of treatment compared with the baseline in the C-3102 group. The relative abundance of genus Fusobacterium was signiffcantly decreased in the C-3102 group at 12 and 24 weeks of treatment compared with the baseline |
| Lambert (2017) | Without reporting the evidence of gut colonization |
| Jafarnejad (2017) | Without reporting the evidence of gut colonization |
| Nilsson (2018) | Without reporting the evidence of gut colonization |
| Vanitchanont (2024) | Without reporting the evidence of gut colonization |
| Gregori (2024) | Without reporting the evidence of gut colonization |
| Harahap (2024) | Without reporting the evidence of gut colonization |
| Zhao (2024) | Probio-M8 co-administration affected the gut microbial interactive correlation network, particularly the short-chain fatty acid-producing bacteria |
| Li (2021) | Without reporting the evidence of gut colonization |
| Zhang (2018) | The results showed that the detection rates of Amycolatopsis ethanolica, Burkholderia oklahomensis, Streptomycesturgidiscabies, and Verrucosiphoramaris in the probiotic group were significantly lower after treatment compared to before treatment |
| Guo (2020) | Without reporting the evidence of gut colonization |

***Supplementary Table S4: Risk of bias assessment for included randomized controlled clinical trials.***

| First author (publication year) | Random sequence generation | Allocation concealment | Blinding of participants and personnel | Blinding of outcome assessment | Incomplete outcome data | Selective reporting | Other sources of bias |
| --- | --- | --- | --- | --- | --- | --- | --- |
| Jansson (2019) | Low | Low | Low | Low | Low | Low | Low |
| Jafarnejad (2017) | Low | Low | Low | Low | Low | Low | Low |
| Lambert (2017) | Low | Low | Low | Low | Low | Low | Low |
| Guo (2020) | Low | Low | Low | Low | Low | Low | Low |
| Nilsson (2018) | Low | Low | Low | Low | Low | Low | Low |
| Takimoto (2018) | Unclear | Low | Low | Low | Low | Low | Low |
| Li (2021) | Low | Unclear | Low | Low | Low | Low | Low |
| Zhang (2018) | Unclear | Unclear | Low | Low | Low | Low | Low |
| Gregori (2024) | Low | Low | Low | Low | Low | Low | Low |
| Zhao (2024) | Unclear | Unclear | Low | Low | Low | Low | Low |
| Vanitchanont (2024) | Low | Unclear | Low | Low | Low | Low | Low |
| Harahap (2024) | Low | Low | Low | Low | Low | Low | Low |

***Supplementary Figure S1A: Risk of bias in seven domains for all included studies***


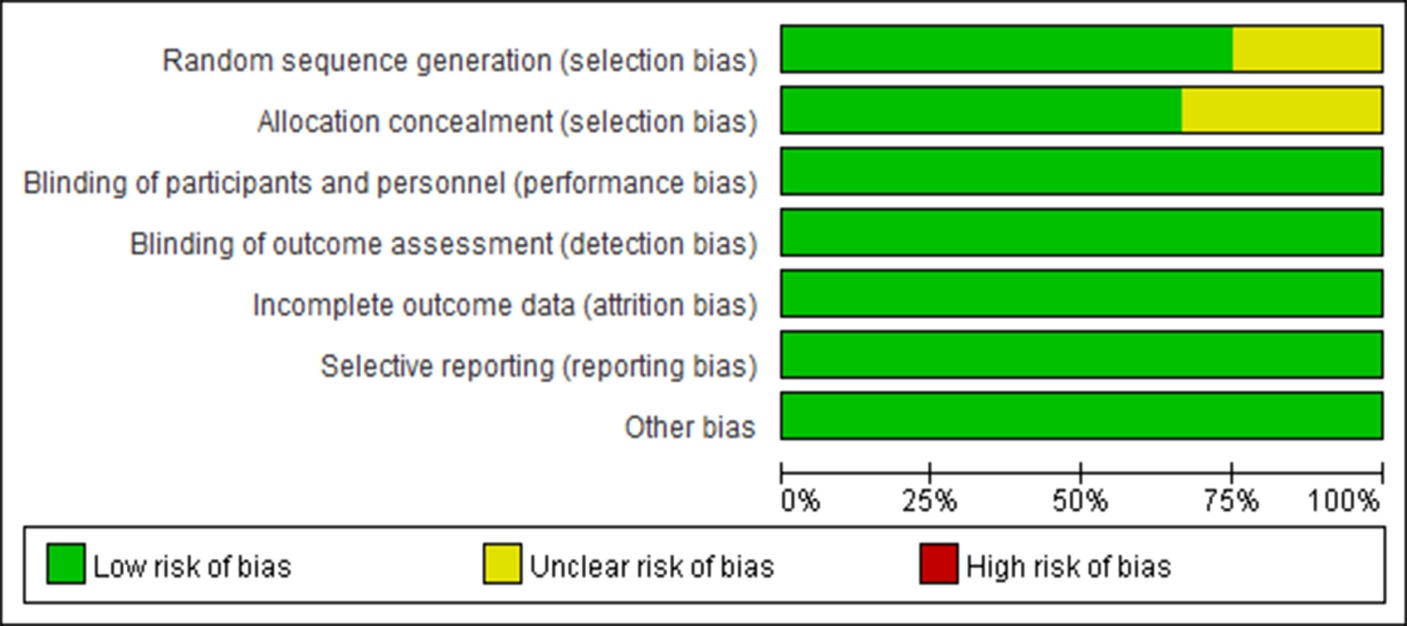


***Supplementary Figure S1B: Risk of bias assessment across seven domains for each included study***


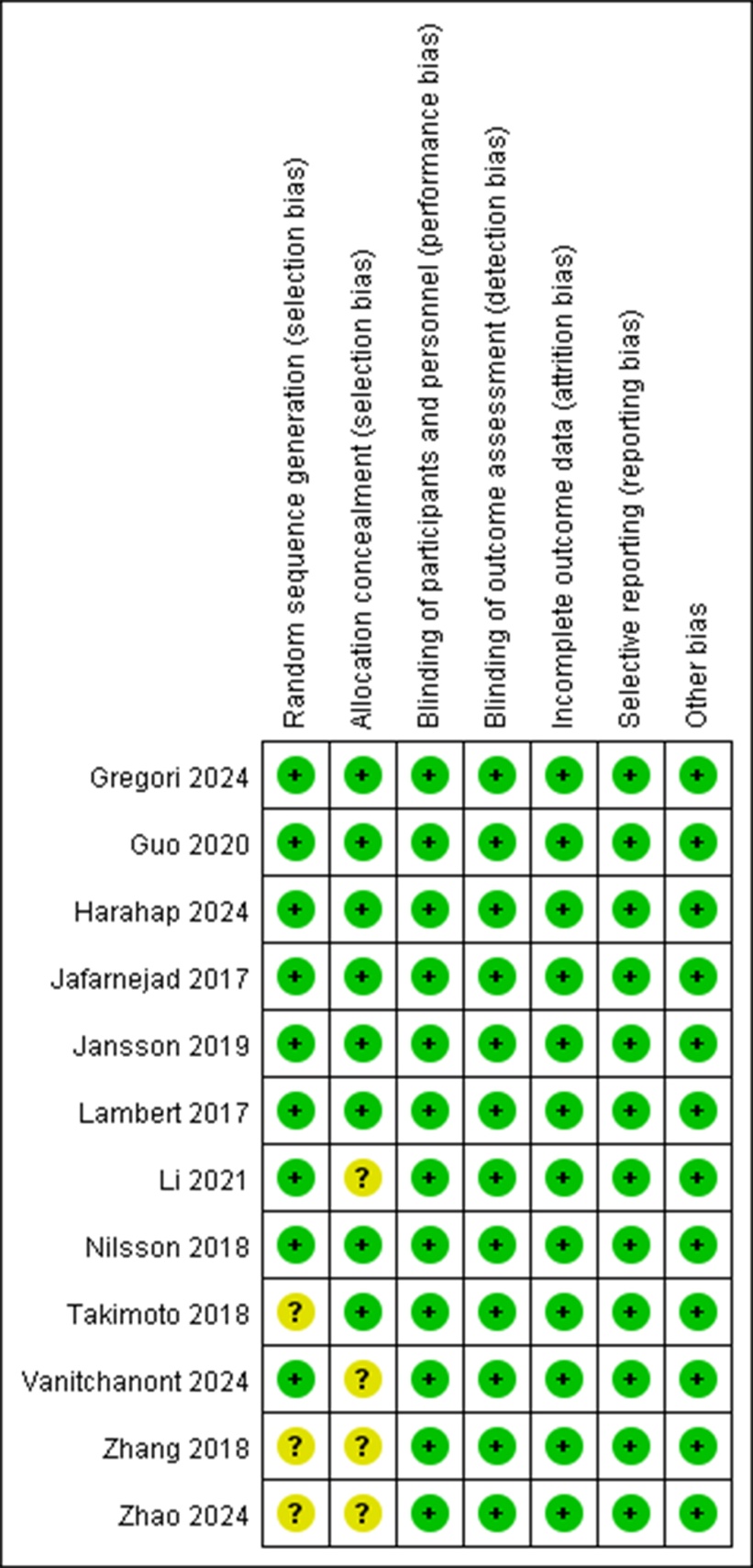


***Supplementary Figure S2A: Meta-regression of age on lumbar spine (1) and hip BMD (2)***

***(1) Meta-regression of age on lumbar spine BMD***

***
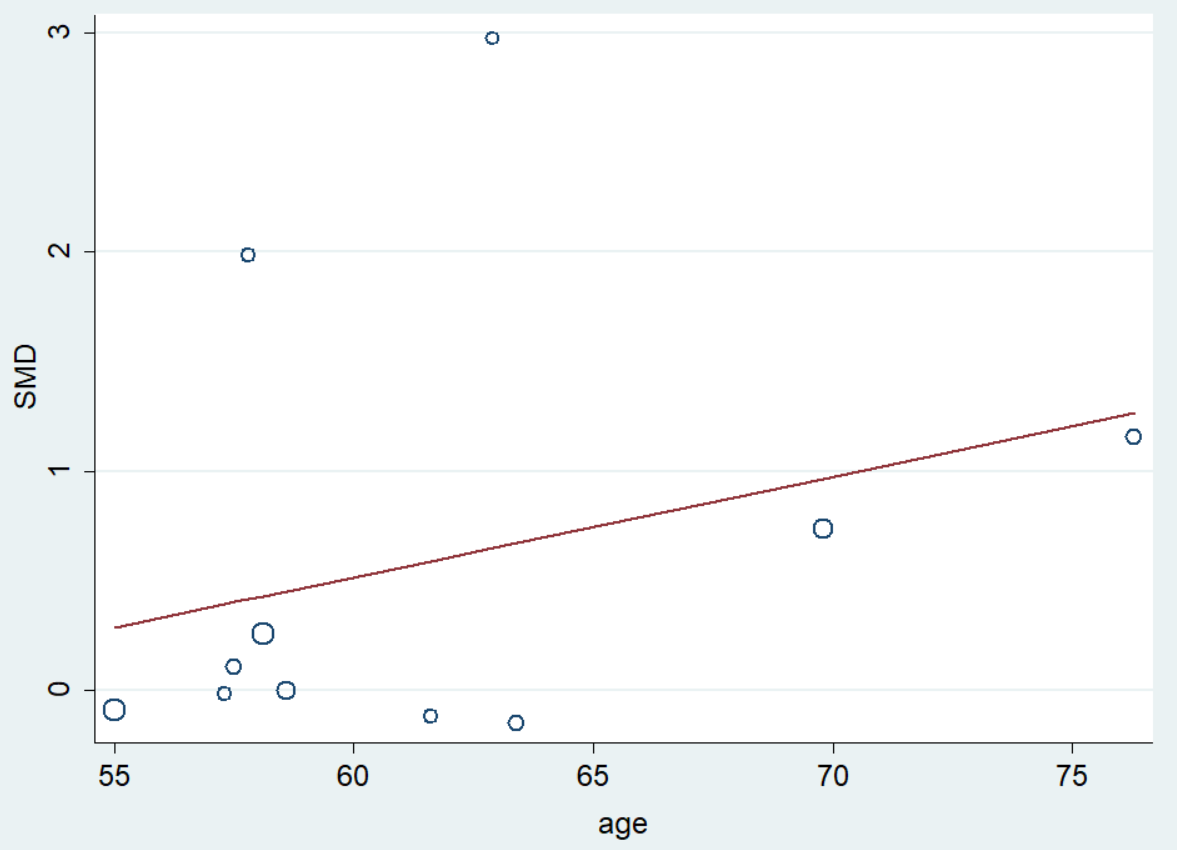
***

***
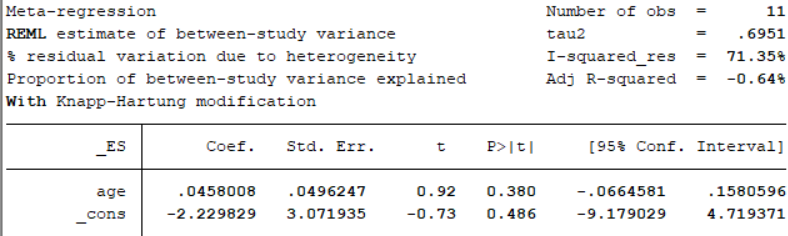
***

***(2) Meta-regression of age on hip BMD***

***
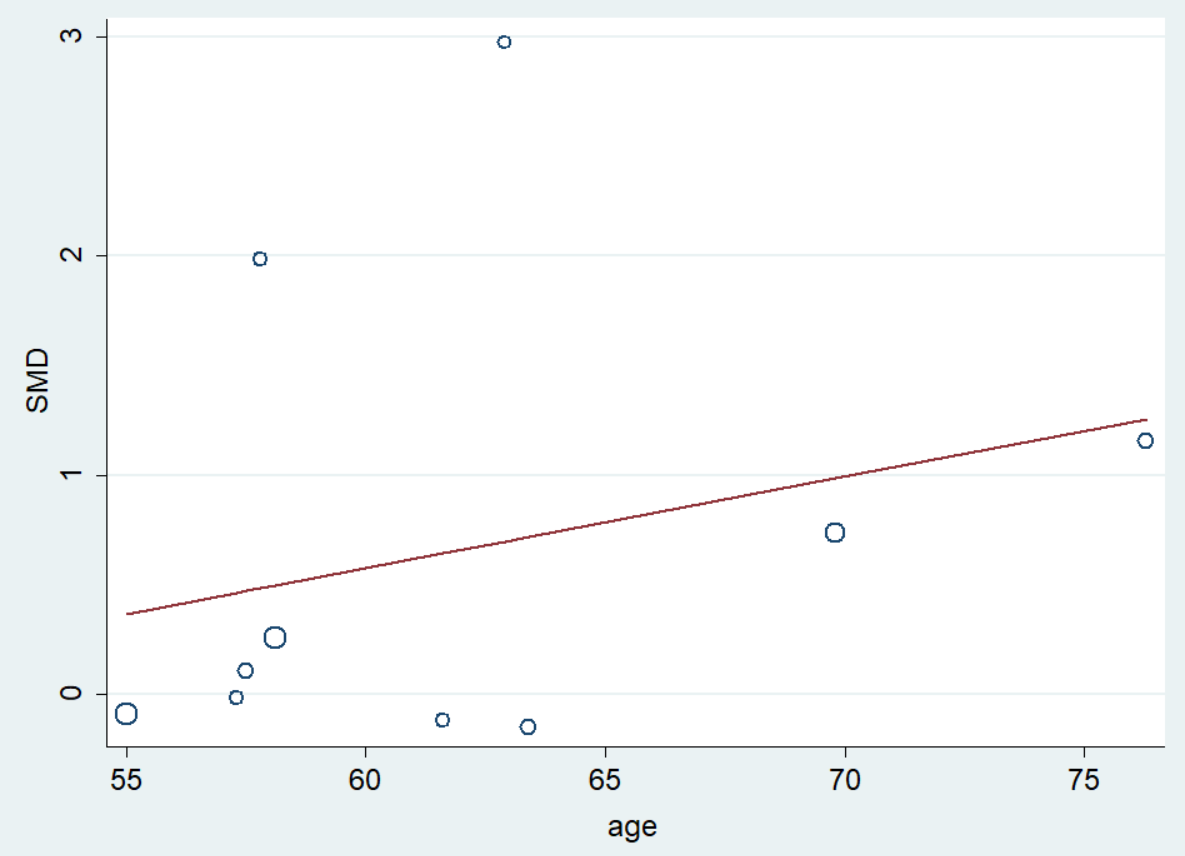
***

***
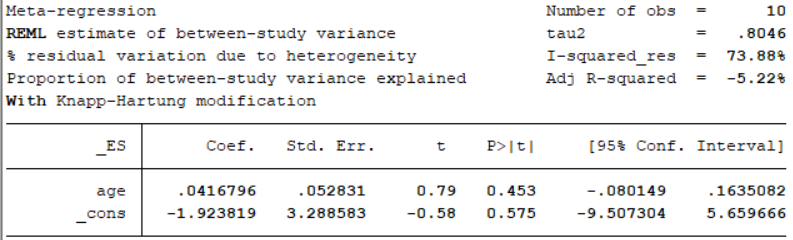
***

***Supplementary Figure S2B: Meta-regression of BMI on lumbar spine (1) and hip BMD (2)***

1. ***Meta-regression of BMI on lumbar spine BMD***

***
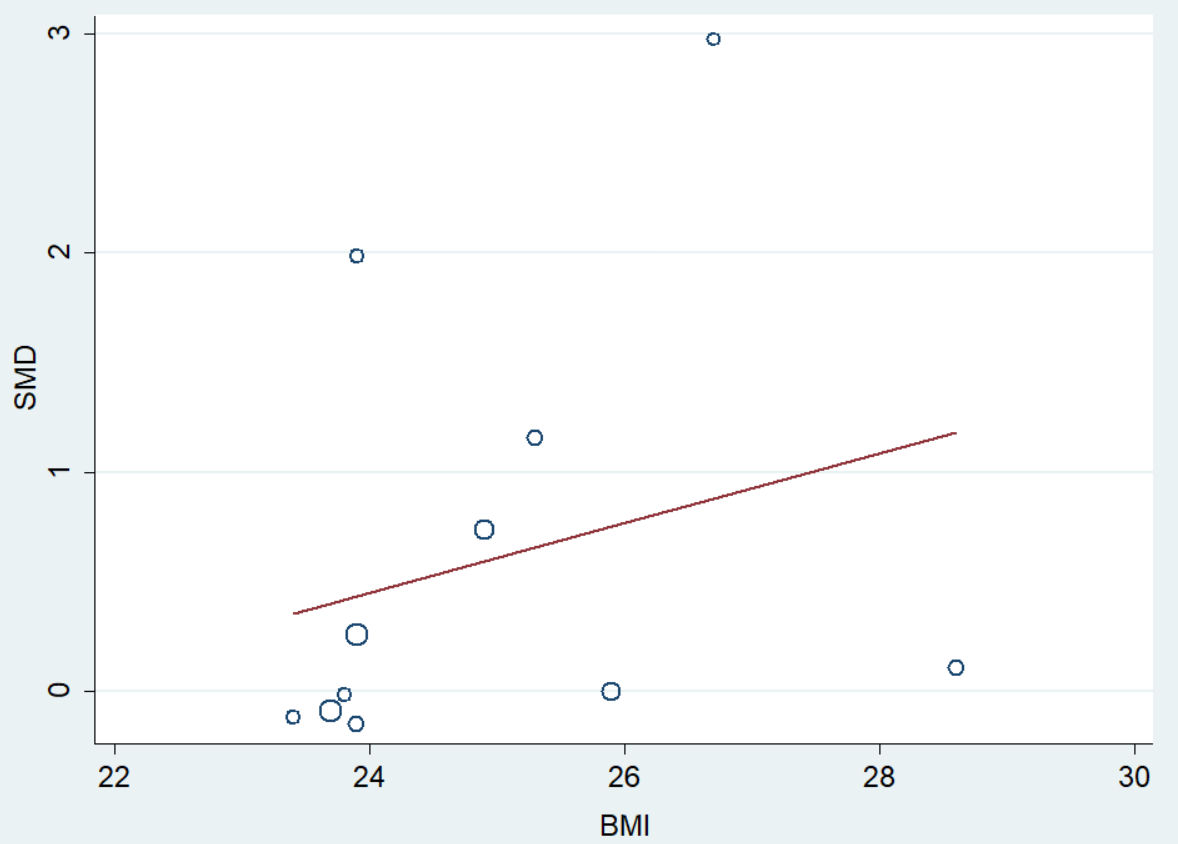
***

***
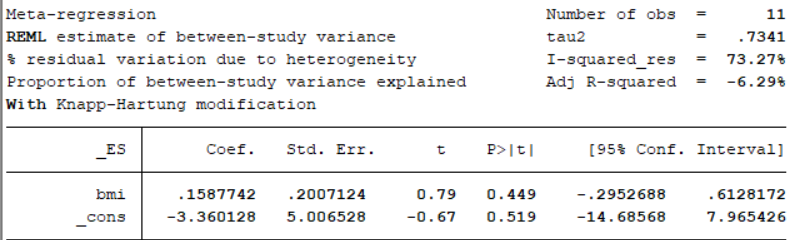
***

1. ***Meta-regression of BMI on hip BMD***

***
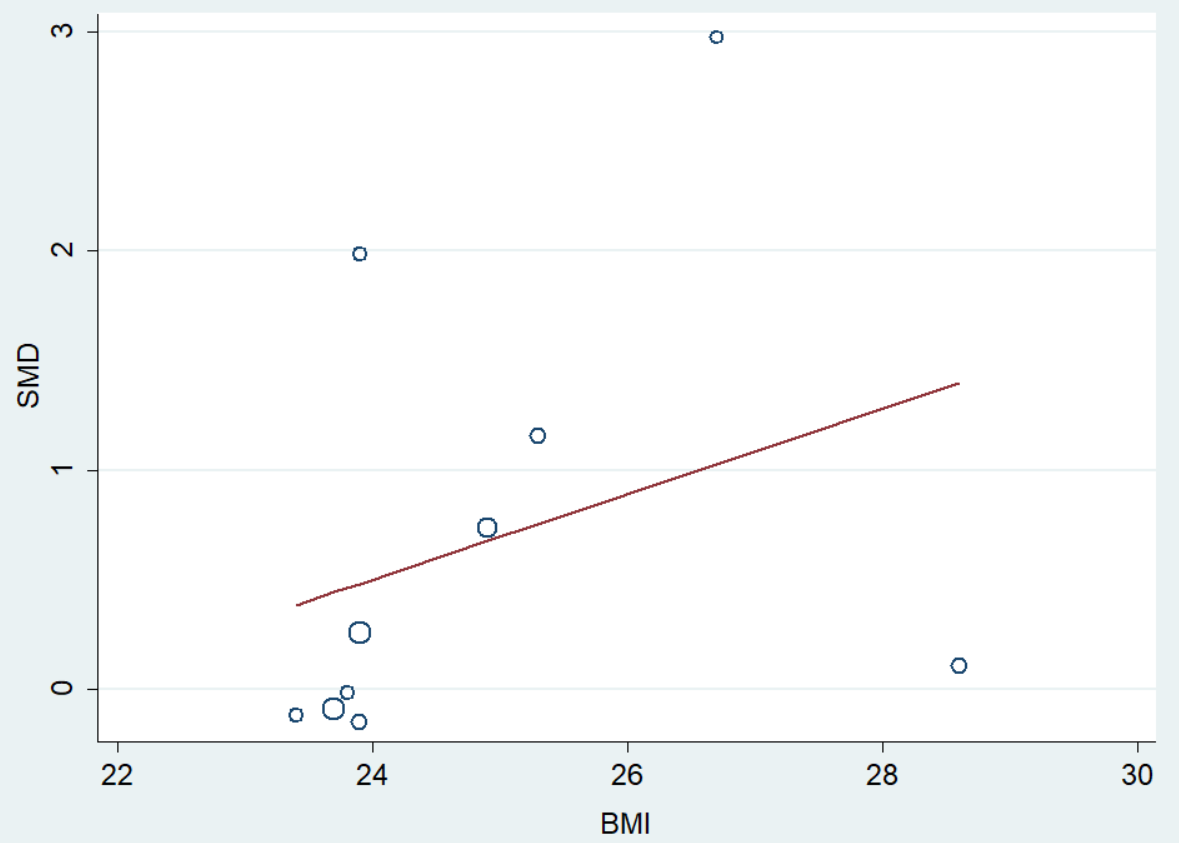
***

***
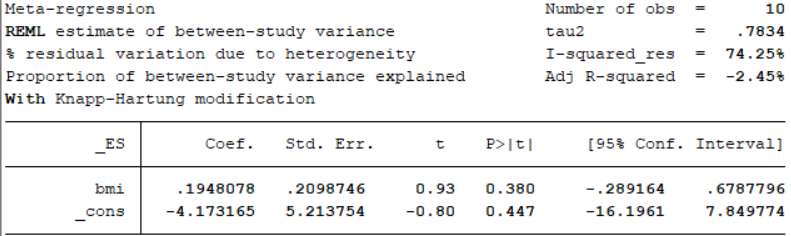
***

***Supplementary Figure S3A: Forest plots depicting the effect of probiotics on osteoprotegerin (OPG)***





***Supplementary Figure S3B: Forest plots depicting the effect of probiotics on receptor activator of nuclear factor-κB ligand (RANKL)***

***
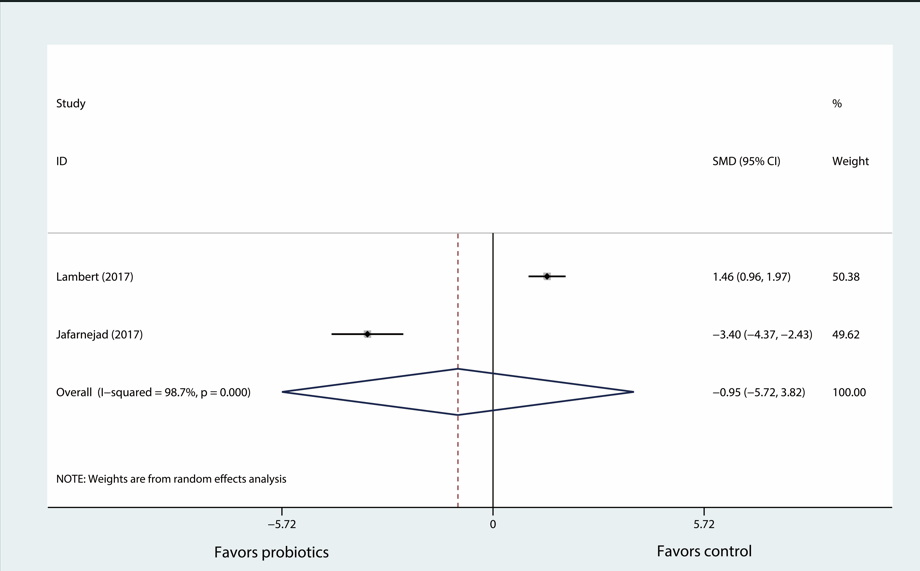
***

***Supplementary Figure S4A: Funnel plot for lumbar spine BMD***


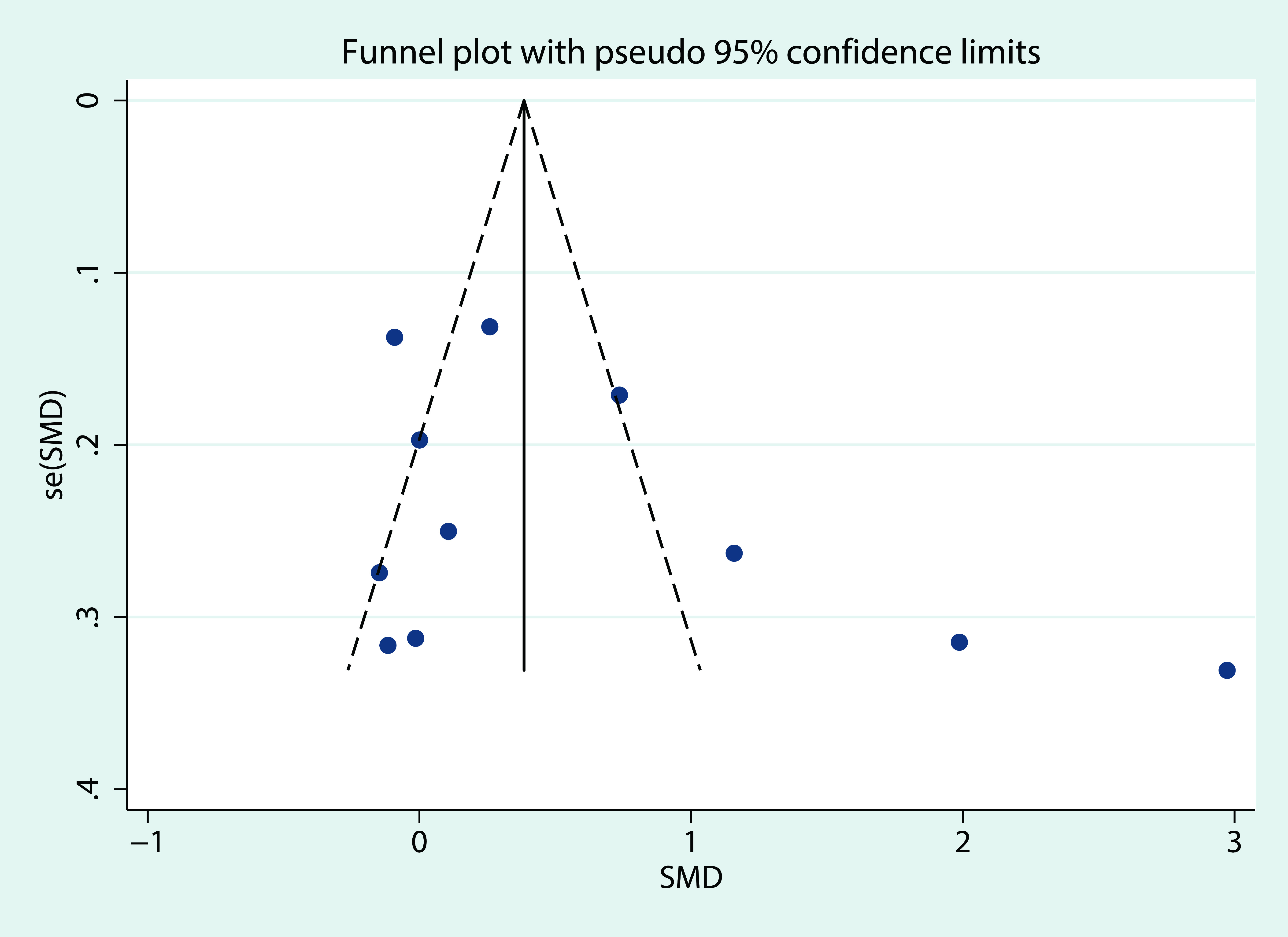


***Supplementary Figure S4B: Funnel plot for hip BMD***


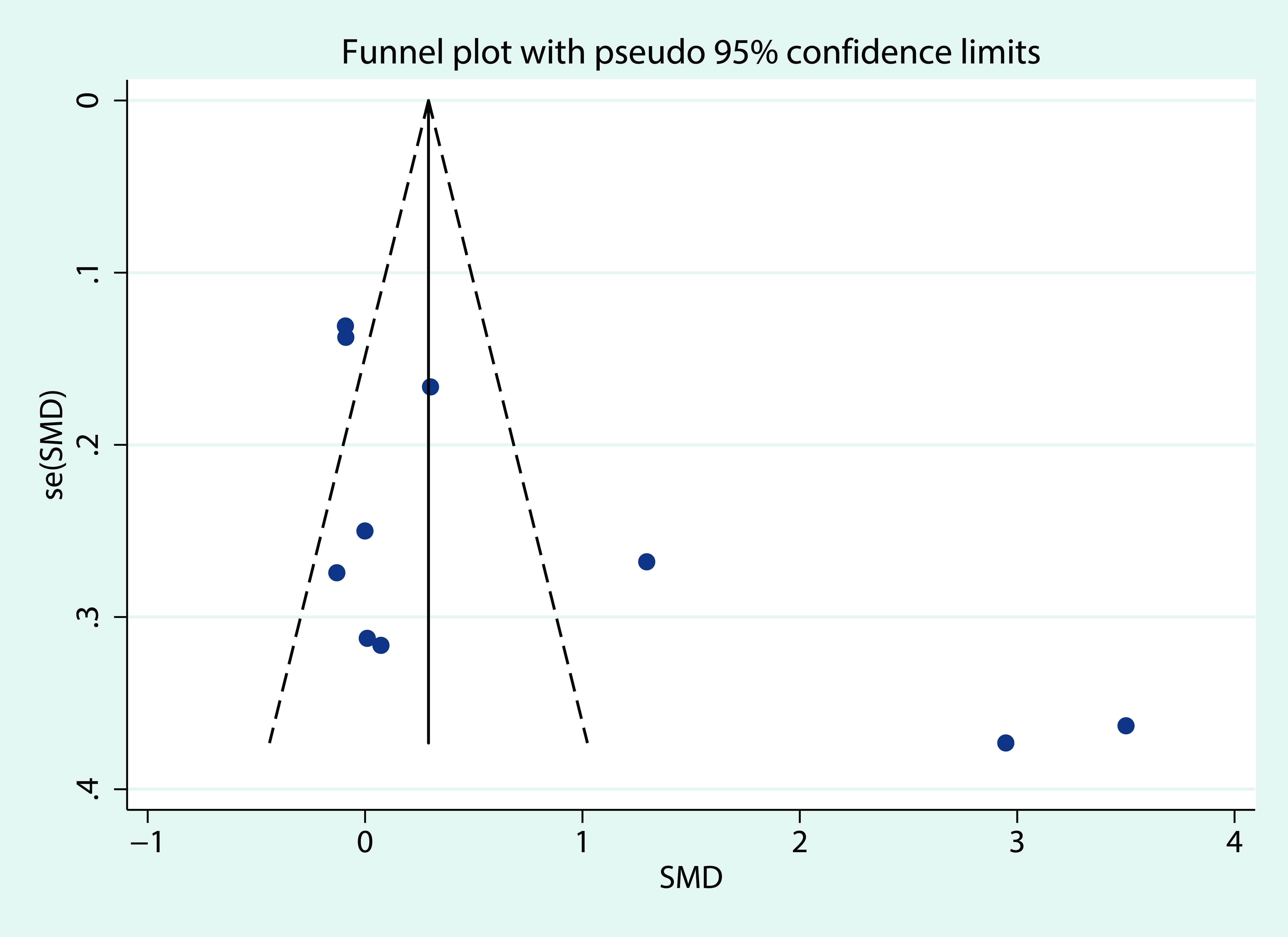


***Supplementary Figure S5A: Egger’s publication bias plot of lumbar spine BMD***

******

***Supplementary Figure S5B: Egger’s publication bias plot of hip BMD***

******

***Supplementary Figure S5C: Publication bias regarding the results of hip BMD after the trim-and-fill method***

******
